# JunB promotes cell invasion, migration and distant metastasis of head and neck squamous cell carcinoma

**DOI:** 10.1186/s13046-016-0284-4

**Published:** 2016-01-12

**Authors:** Hiroshi Hyakusoku, Daisuke Sano, Hideaki Takahashi, Takashi Hatano, Yasuhiro Isono, Shoko Shimada, Yusuke Ito, Jeffrey N. Myers, Nobuhiko Oridate

**Affiliations:** Department of Biology and Function in Head and Neck, Yokohama City University Graduate School of Medicine, Yokohama, Japan; Department of Otorhinolaryngology - Head and Neck Surgery, Yokohama City University, School of Medicine, 3-9 Fukuura, Kanazawa-ku, Yokohama, 236-0004 Japan; Department of Urology, Yokohama City University Graduate School of Medicine, Yokohama, Japan; Department of Head and Neck Surgery, The University of Texas M. D. Anderson Cancer Center, Houston, Texas USA

**Keywords:** JunB, AP-1, Head and neck squamous cell carcinoma (HNSCC), Distant metastasis, CRISPR/Cas9-mediated knockout

## Abstract

**Background:**

While treatment failure in cases of head and neck squamous cell carcinoma (HNSCC) frequently takes the form of locoregional recurrences and distant metastasis, our understanding of the mechanisms of metastasis in HNSCC is limited. We initially performed the upstream and key nodes analysis together with whole gene microarray analysis characterized by distant metastatic potential *in vivo* with HNSCC cell lines and identified JunB, a member of the activator protein-1 (AP-1) family, as a key molecule in the regulation of the pathways related to distant metastasis in HNSCC. We have therefore tested the hypothesis that JunB plays a crucial role in distant metastasis in HNSCC.

**Methods:**

To study the role of JunB on metastatic potential of HNSCC, small interfering RNA (siRNA)-mediated knockdown and clustered regularly interspaced short palindromic repeats (CRISPR)/CRISPR-associated protein 9 (cas9) system (CRISPR/Cas9)-mediated knockout of JunB in HNSCC cells were established and the abilities of cell invasion and migration *in vitro* were examined. The efficacy of knockout of JunB was also examined using an experimental lung metastatic mouse model of HNSCC. In addition, to study if the role of JunB in HNSCC cell migration and invasiveness is related to epithelial-to-mesenchymal transition (EMT), cell morphology and expression of mesenchymal or epithelial marker on siRNA mediated JunB knockdown in HNSCC cells were examined with or without TGF-β stimulation.

**Results:**

siRNA knockdown and sgRNA knockout of JunB in metastatic HNSCC cells significantly suppressed both cell invasion and migration *in vitro*. In addition, the knockout of JunB in metastatic HNSCC cells significantly repressed the incidence of lung metastases and prolonged the survival *in vivo*. However, we did not observe any change in cell morphology with the down-regulation of mesenchymal markers and up-regulation of epithelial markers in response to siRNA-mediated JunB knockdown in HNSCC cells.

**Conclusion:**

These results suggested that JunB could play an important role in promoting cell invasion, migration and distant metastasis in HNSCC via pathways other than EMT and that the down-regulation of JunB may become an effective strategy for patients with invasive HNSCC.

**Electronic supplementary material:**

The online version of this article (doi:10.1186/s13046-016-0284-4) contains supplementary material, which is available to authorized users.

## Background

Head and neck squamous cell carcinoma (HNSCC) is the sixth most frequently diagnosed cancers in the world [1]. The survival of patients with HNSCC has not dramatically improved over the past several decades despite advances in multidisciplinary treatment [2, 3]. This is because of many newly diagnosed HNSCC patients present with advanced stage disease at diagnosis, and partly due to our inability to control and our poor understanding of the regional and distant spread of this disease. In fact, most treatment failure in cases of HNSCC is due to locoregional recurrence or distant metastatic disease [4, 5]. Thus, it is still urgent to advance our understanding of the mechanisms of the progression and metastasis of HNSCC in order to improve the survival outcome for patients with HNSCC.

The transcription factor AP-1 (activator protein 1), one of the major effectors activating gene transcription, is a heterodimeric protein composed of Fos family (Fos, FosB, FosL1 and FosL2), Jun family (Jun, JunB, and JunD), Atf (activating transcription factor) and Maf (musculoaponeurotic fibrosarcoma) proteins [6, 7]. Phosphorylation of Fos and Jun or extracellular stimuli such as cytokines, stress, infection, and growth factors inducing the expression of Fos and Jun, the main AP-1 proteins in mammalian cells, can activate the AP-1 pathway. The activated AP-1 complex then binds to a consensus DNA sequence in the promoter region to regulate AP-1 target genes expression thus playing an important role in a number of cellular processes, including proliferation, differentiation, apoptosis, cell migration, and transformation [8]. While some AP-1 proteins have been reported to have tumor suppressor activity, AP-1 is well known to have oncogenic activity [6, 9, 10]. In fact, AP-1 activation in epithelial cells has been reported to be required for SCC transformation in a transgenic mouse model [11], and to promote metastasis in SCC [12]. Among the AP-1 family, Jun and FosL1 have also been reported to promote invasion via epithelial-to-mesenchymal transition (EMT) [9]. Thus, the activation of AP-1 has been reported to play a critical role in the invasive growth and metastasis of human cancers, although the significance of AP-1 in metastasis in HNSCC is not yet fully understood.

In this study, we initially characterized the distant metastatic potential *in vivo* using 26 different HNSCC cell lines in an experimental lung metastatic mouse model with tail vein injection of HNSCC. A whole gene microarray was performed with 8 selected HNSCC cell lines, and upstream and key node analysis was then used to investigate the upstream key molecules involved in the mechanisms of distant metastasis in HNSCC. The AP-1 family was identified as the key molecules regulating the pathways related to distant metastasis in HNSCC. We therefore hypothesize that the AP-1 family plays a crucial role in inducing cell invasion, migration and distant metastasis in HNSCC. In the present study, we show that the small interfering RNA (siRNA)-mediated knockdown and clustered regularly interspaced short palindromic repeats (CRISPR)/CRISPR-associated protein 9 (cas9) system (CRISPR/Cas9 [13, 14])-mediated knockout of JunB in HNSCC cells significantly inhibited both invasion and migration *in vitro*, as well as lung metastasis *in vivo*.

## Methods

### Cell lines

Information on and appropriate growth media for the 26 HNSCC cell lines are shown in Additional file 1: Table S1. All cells were authenticated by short tandem repeat genotyping as described previously [15, 16]. Adherent monolayer cultures were maintained on plastic and incubated at 37 °C and 5 % CO_2_.

### Animals and maintenance

Athymic nude mice, aged 7–8 weeks, were purchased from the animal production area of the National Cancer Institute-Frederick Cancer Research and Development Center (Frederick, MD) and Oriental Yeast (Tokyo, Japan). The mice were housed and maintained in laminar flow cabinets under specific pathogen-free conditions and used in accordance with the NIH and AERI-BBRI Animal Care and Use Guidelines under protocols approved by the Institutional Animal Care Use Committee of the University of Texas M.D. Anderson Cancer Center (Houston, TX) and Yokohama City University School of Medicine (Yokohama, Japan).

### Experimental lung metastatic mouse model of HNSCC

The lung metastatic potential of the 26 HNSCC cells was examined by a tail vein metastatic assay as described previously [17]. Briefly, 1.0 × 10^6^ HNSCC cells in a volume of 200 μL were injected into the lateral tail vein using a 27-gauge needle. Eight to 11 mice were injected with each cell line. Mice were euthanized using carbon dioxide asphyxiation when they lost more than 15 % of their pre-injection body weight or at 90 days after cell injection. Necropsy was performed and lungs were harvested and then weighed. Each lung was fixed in formalin and embedded in paraffin for hematoxylin and eosin (H&E) staining. The presence of lung metastases was evaluated by light microscopy.

### Microarray analysis and upstream and key node analysis with ExPlain™

For the microarray analysis, total RNA was extracted from 8 HNSCC cell lines (metastatic; HN30, YCU-T892 and KCC-T871, non-metastatic; Detroit562, PE/CA-PJ34, KCC-M871, YCU-MS861, and YCU-M862) as described previously [18]. Whole genome gene profiling was performed using a SurePrint G3 Human GE 8 × 60 K Microarray (Agilent Technologies, Santa Clara, CA, USA). The raw data were deposited into the Gene Expression Omnibus (GEO, accession number: GSE67275).

Principal component analysis (PCA) was then performed based on all the probe sets utilized in our microarray analysis. The 8 principal components were checked for differences between metastatic lines and non-metastatic lines, and statistical significance was assessed by unpaired *t*-test. Genes with an absolute fold change value > 5.5 and *P* < 0.05 were selected for further analysis. Upstream and key nodes analysis with ExPlain™ (www.biobase.de) was then performed to investigate upstream key molecules involved in the mechanisms for distant metastasis in HNSCC as described previously [19, 20]. Each key node is assigned a score based on its connectivity to the pathways. Molecules with a higher score can be considered to be key factors in the regulation of the pathways.

### Western blotting analysis

Western blot analyses were performed to determine the expression of FosL1, c-Jun and JunB in HNSCC cell lines as described previously [21]. Antibodies were purchased from the following sources and used at the indicated dilutions: FosL1 (1:1000; Cell Signaling Technologies, Danvers, MA), c-Jun (1:1000; Cell Signaling Technologies), JunB (1:1000; Cell Signaling Technologies), E-cadherin (1:1000; Cell Signaling Technologies), N-cadherin (1:1000; Santa Cruz Biotechnology, Dallas, TX) Vimentin (1:1000; Santa Cruz Biotechnology), and α-Tubulin (1:1000; Cell Signaling Technologies). To study TGF-β1-mediated EMT, HNSCC cells were serum-starved overnight and then treated with or without 2 ng/mL transforming growth factor-β1 (TGF-β1; R&D Systems, Minneapolis, MN) for 24 h.

### siRNA-mediated knockdown of JunB in HNSCC cells

HNSCC cell lines were transiently transfected with scrambled control or two independent siRNAs for *JUNB* (siRNA IDs: 7661 and s7662) (Life Technologies, Gaithersburg, MD) using Lipofectamine RNAiMAX (Life Technologies) according to the manufacturer’s instructions. JunB protein expression levels in the JunB knockdown cells were compared with that of cells transfected with a negative siRNA control by Western blotting.

### CRISPR/cas9-mediated knockout of JunB in HNSCC cells

The cloning of top and bottom oligonucleotides, annealing and ligation were performed using a GeneArt CRISPR Nuclease Vector with a CD4 Enrichment Kit (Life Technologies). KCC-T871 cells were transfected with single-guide RNA (sgRNA) for two independent specific sequences in *JUNB* (JunB#1 and JunB#2) or nonspecific sgRNA using Lipofectamine 3000 (Life technologies) and Amaxa Nucleofector 2b (Lonza, Basel, Switzerland). Electroporation/nucleofection was performed using a Cell Line Nucleofector kit V (Lonza) and the Nucleofector program T-030. Control and *JUNB* oligonucleotides are shown in Table 1. Single colonies were isolated using a Dynabeads CD4 Positive Isolation Kit (Life technologies) for further passaging.

**Table 1:** Sequences of CRISPR sgRNA and confirming primers used in this study

Name	sgRNA sequence (5′–3′)
Control (forward)	CATTTCTCAGTGCTATAGAGTTTT
Control (reverse)	TCTATAGCACTGAGAAATGCGGTG
*JUNB*#1 (forward)	GTCTCTCAAGCTCGCCTCTTGTTTT
*JUNB*#1 (reverse)	AAGAGGCGAGCTTGAGAGACCGGTG
*JUNB*#2 (forward)	GCATCAAAGTGGAGCGCAAGGTTTT
*JUNB*#2 (reverse)	CTTGCGCTCCACTTTGATGCCGGTG
	confirming primers
*JUNB*#1 (forward)	TGGAACAGCCCTTCTACCAC
*JUNB*#1 (reverse)	TGCTGAGGTTGGGTGTAAACG
*JUNB*#2 (forward)	CGACCACCATCAGCTACCTC
*JUNB*#2 (reverse)	AGAAGGCGTGTCCCTTGAC

The confirmation of the genome editing was performed using a GeneArt Genomic Cleavage Detection kit (Life technologies) with the primers shown in Table 1. PCR products were visualized by means of an E-Gel Safe Imager on E-GeL EX 2 % agarose (Life technologies). JunB protein expression levels in sgRNA-transfected or control cells were confirmed by Western blotting.

### Invasion assay

*In vitro* tumor cell invasion was examined using Corning Matrigel Invasion Chambers (Corning life science, Corning, NY). Briefly, 5 × 10^4^ of KCC-T871 cells or 1 × 10^5^ of HN30 cells infected with scramble or *JUNB* siRNA in serum-free medium were plated in the upper chamber and incubated with medium containing 10 % fetal bovine serum (FBS) in the bottom of the chamber for 22 h. Invaded cells were then stained with giemsa solution (WAKO, Japan) and counted in all fields. The experiment was repeated three times.

### Scratch assay

One million KCC-T871 or HN30 cells infected with scrambled or *JUNB* siRNA, or with sgControl or *JUNB* sgRNA were seeded in 24-well plates and incubated with medium containing 10 % FBS. Once confluent, a horizontal wound was made in the cell layer of each well using a 200-μL pipette tip and images were captured at 0 h and 9 h post-wound for KCC-T871 and 15 h for HN30 cells. The percentage of the wound area remaining open was measured to assess the amount of movement during wound closure. The experiment was repeated three times.

### Cell viability assay

Cells were seeded on 96-well microplates at the concentration of 1.0 × 10^3^ cells per well and cultured at 37 °C in 5 % CO_2_, and then incubated for 24, 48, 72 or 96 h. Cell viability was evaluated by Cell Counting Kit-8 (CCK-8) assay (Dojindo Laboratories, Kumamoto, Japan), in which the absorbance at OD 450 nm was measured using a microplate reader (BioRad, Model 680, USA).

### Experimental lung metastatic mouse model with KCC-T871/crControl and KCC-T871/*JUNB/*KO#1 cells

The lung metastatic potential of KCC-T871 cells transfected with the sgRNA control or sgRNA *JUNB*#1 was examined using a tail vein metastatic assay. A total of 7 × 10^5^ cells were injected into the lateral vein as described previously [17]. Fourteen or 16 mice were prepared for each cell line. Mice were euthanized using carbon dioxide asphyxiation when they lost more than 15 % of their pre-injection body weight or at 120 days after cell injection. The presence of lung metastasis was confirmed by H&E staining.

The animal experiment was repeated with 6 mice for the control and 8 mice for the sgRNA-mediated JunB knockout group. In the repeated experiment, mice were euthanized at 78 days after cell injection, and lungs were then weighed to evaluate for the presence of metastasis by light microscopy. To quantify the development of lung metastasis in the animal model, we calculated the average ratio of the area displaying metastatic cells in the lung to the total area of the lung in an entire field. Three lung slides were prepared for each mouse in this analysis.

### Statistical analysis

Potential correlations between the incidence of lung metastasis (%) and median survival time in the experimental lung metastasis model were analyzed using Pearson’s correlation. Survival was analyzed by the Kaplan-Meier method and compared using log-rank tests. The results of the migration and invasion assay were compared using a paired 2-tailed *t* test. Fisher’s exact test was used to compare the incidences of lung metastasis. Quantitative data related to median lung weight and the area displaying metastatic cells in the lung were compared using an unpaired 2-tailed *t* test. Statistical analyses were performed with GraphPad Prism version 6.05 (GraphPad Software, La Jolla, CA). For all comparisons, *P* < 0.05 was considered statistically significant.

## Results

### The distant metastatic potential of 26 HNSCC lines in an experimental lung metastatic mouse model

**Fig. 1:**
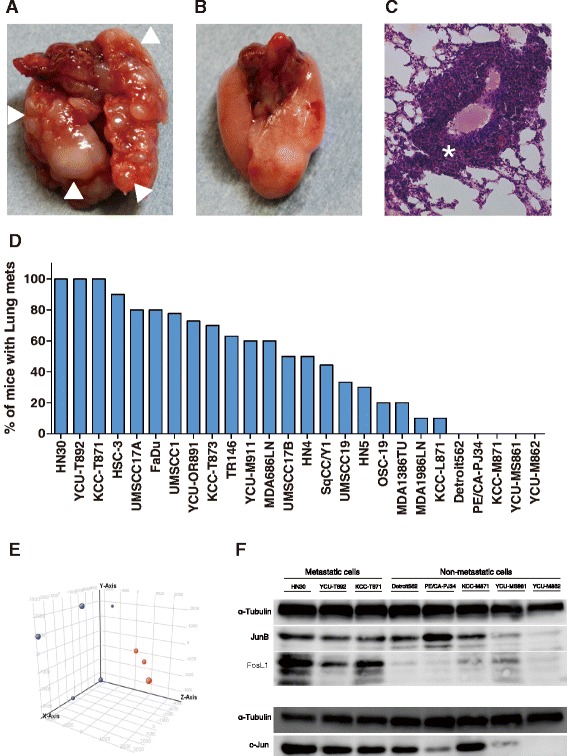
The distant metastatic potential of 26 HNSCC lines in the experimental lung metastatic mouse model. **a** Lung metastasis in the experimental lung metastatic mouse model of HNSCC. (△ metastatic lesion). **b** No metastasis was observed in the lungs of the mouse model. **c** H&E staining of lung metastasis in the experimental lung metastatic mouse model of HNSCC (* metastatic lesion). **d** Incidence of lung metastasis for 26 cell lines in the experimental lung metastatic mouse model of HNSCC. Three HNSCC cell lines (HN30, KCC-T871, YCU-T892) established 100 % lung metastases, while 5 cell lines (Detroit562, PE/CA-PJ34, KCC-M871, YCU-MS861, YCU-M862) did not establish lung metastasis in any of the mice injected. **e** The results of principle component analysis (PCA). PCA was performed based on the expression profiles of samples. The first 3 PCAs for 8 HNSCC cells were plotted. : Metastatic lines (HN30, KCC-T871, YCU-T892), : Non-metastatic lines (Detroit562, PE/CA-PJ34, KCC-M871, YCU-MS861, YCU-M862). **f** Expression of AP-1 family proteins in HNSCC cells by Western blotting. The lung metastatic HNSCC cell lines (HN30, YCU-T892 and KCC-T871) showed higher levels of c-Jun, FosL1 and JunB expression than did the non-metastatic HNSCC cell lines. α-Tubulin was used as an internal control

Twenty-six HNSCC cell lines showed a wide spectrum of distant metastatic potential *in vivo*. We found that 21 (80.8 %) of the 26 HNSCC cell lines produced lung metastases (Fig. 1d, Additional file 2: Table S2). Three HNSCC lines (HN30, KCC-T871, YCU-T892) established 100 % lung metastases in an experimental lung metastatic mouse model of HNSCC (Fig. 1a, c), while five HNSCC cell lines (Detroit562, PE/CA-PJ34, KCC-M871, YCU-MS861, YCU-M862) did not establish lung metastasis in any of the mice injected (Fig. 1b). Survival curves for mice injected with each of the 26 HNSCC cell lines are shown in Additional file 3: Figure S1. The median survival time ranged from 43.5 to 90 days. We found an inverse correlation between the incidence of lung metastasis (%) and median survival time (*r* = –0.5195, *P* = 0.0015) (Additional file 4: Figure S2).

### Microarray analysis and upstream and key node analysis

A clear separation in the 3 principal component analysis (PCA) was observed between three metastatic HNSCC cell lines (HN30, KCC-T871, YCU-T892) and five non-metastatic HNSCC cell lines (Detroit562, PE/CA-PJ34, KCC-M871, YCU-MS861, YCU-M862) (Fig. 1e). Differentially expressed genes showing statistically significant up- or down-regulations in expression between the metastatic and non-metastatic HNSCC cell lines are shown in Table 2.

**Table 2:** Top 32 lists of expressed genes showing statistically between metastatic and non-metastatic HNSCC cells

ProbeName	GeneSymbol	Fold change ([100 %] vs [0 %])	Absolute Fold change ([100 %] vs [0 %])
A_24_P393571	GDA	266.48	266.48
A_33_P3238166	PXDN	224.54	224.54
A_33_P3271455	PXDN	200.54	200.54
A_33_P3846653	KRT19P2	−160.22	160.22
A_23_P66798	KRT19	−152.13	152.13
A_23_P36658	MGST1	−148.41	148.41
A_33_P3342375	MAGEA6	114.26	114.26
A_23_P501754	CSF3	106.48	106.48
A_23_P89431	CCL2	−93.74	93.74
A_23_P23155	AJAP1	91.15	91.15
A_23_P148255	MAGEA2B	88.90	88.90
A_23_P383258	GDA	86.16	86.16
A_23_P29594	RPL39L	−68.22	68.22
A_23_P29655	C3orf14	65.82	65.82
A_33_P3307363	LPHN2	−60.56	60.56
A_23_P122924	INHBA	54.27	54.27
A_32_P101031	LYPD1	−45.41	45.41
A_33_P3284129	LYPD1	−45.37	45.37
A_23_P518	VTCN1	−45.19	45.19
A_33_P3687198	LOC283454	−43.10	43.10
A_23_P135257	PRSS3	38.15	38.15
A_23_P310274	PRSS2	36.77	36.77
A_23_P43197	CALB1	−36.67	36.67
A_23_P70307	SMOC2	36.55	36.55
A_21_P0009342	LOC645638	−35.05	35.05
A_33_P3222424	CSF3	34.90	34.90
A_23_P218111	SERPINA1	34.12	34.12
A_33_P3240078		32.19	32.19
A_23_P415021	METTL7A	−31.60	31.60
A_19_P00805833	LOC100287628	−31.45	31.45
A_33_P3352557	DMRTA2	−31.07	31.07
A_23_P87709	PLBD1	−30.70	30.70

One hundred sixty four genes with an absolute fold change value (FC) >5.5 and *P* < 0.05 were detected. Top 32 genes with an absolute FC > 30 were listed

To better understand the mechanisms underlying the gene expression findings, the microarray data were analyzed in the context of complex regulatory networks. One hundred and sixty-four genes with an absolute fold change value (FC) >5.5 and *P* < 0.05 (unpaired *t*-test) were selected, and the data were then loaded into the ExPlain™ pathway search tool and key nodes were searched in the upstream pathways.

One hundred and ninety-seven genes were identified for the candidate genes as key factors in the regulation of pathways related to distant metastasis in HNSCC. A list of 20 genes with a score ≥11 according to the ExPlain™ tool is shown Table 3. The results, which show several AP-1 family genes such as Fos, JunB and FosL1 as having high scores, led us to hypothesize that the AP-1 family of transcription factor plays a crucial role in inducing cell invasion, migration and distant metastasis in HNSCC.

**Table 3:** Top 20 lists of upstream key molecules in lung metastatic versus non-metastatic HNSCC cells

Key molecules	score
FOS	26
JUNB	23
FOSL1	21
PPARG	21
IRF8	20
IRF1	19
CBP	18
IRF4	18
ISGF3G	17
IKK-beta	16
FOXA2	14
IRF7	14
NR3C1	14
JUND	13
Src-isoform1	13
IRF2	12
beta-catenin	11
c-Myc-isoform1	11
MKK4beta	11
p38alpha	11

Microarray data were loaded into the ExPlain™ pathway search tool and key nodes were searched in the upstream pathways. Each key node is assigned a score based on its connectivity of the pathways. Molecules with higher score could be considered as key factors regulating the pathways related with distant metastasis of HNSCC

### Expression of AP-1 family proteins in HNSCC cells

The expression levels of c-Jun, FosL1 and JunB in metastatic HNSCC cells (HN30, KCC-T871, YCU-T892) and non-metastatic HNSCC cells (Detroit562, PE/CA-PJ34, KCC-M871, YCU-MS861, YCU-M862) were analyzed by Western blotting as shown in Fig. 1f. The lung metastatic HNSCC cell lines showed higher levels of c-Jun, FosL1 and JunB expression than did the non-metastatic HNSCC cell lines. Although the difference in JunB expression between the metastatic and non-metastatic HNSCC cell lines was slight, we decided to clarify the roles of JunB in regulating the pathways related to distant metastasis in HNSCC based on the high scores observed for JunB in the upstream and key node analysis for the current dataset (distant metastatic vs. non-metastatic) and the regional metastatic vs. non-metastatic data set (data not shown).

### siRNA knockdown and sgRNA knockout of JunB in metastatic HNSCC cells suppresses tumor invasion and migration

To determine whether JunB promoted invasion and migration in HNSCC cells, we depleted JunB in metastatic HNSCC cell lines (KCC-T871 and HN30), and performed invasion and scratch assays.

**Fig. 2:**
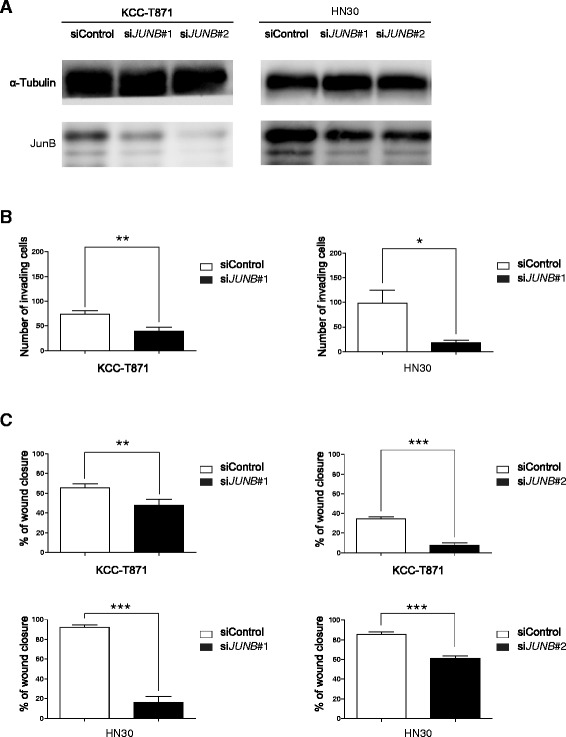
siRNA knockdown of JunB in metastatic HNSCC cells suppresses tumor invasion and migration. **a** Western blotting analysis of KCC-T871 and HN30 cells transfected with scramble or *JUNB* siRNAs (#1 and #2)*.* **b** siRNA knockdown of JunB in HN30 and KCC-T871 cells inhibited cell invasion. Columns, mean number of cells; bars, SEM. *P < 0.05, **P < 0.01. **c** siRNA knockdown of JunB in HN30 and KCC-T871 cells inhibited cell migration. Columns, mean number of cells; bars, SEM. **P < 0.01, ***P < 0.001

KCC-T871 and HN30 were transfected with siControl or two independent siRNAs for *JUNB* (#1 and #2) and the knockdown was then confirmed by Western blotting (Fig. 2a). The invasion assay revealed a 45.9 % reduction in invasion potential for KCC-T871/si*JUNB*#1 cells (39.8 ± 7.7) compared to scrambled siRNA control (73.5 ± 7.0, *P* = 0.0003) and an 81.2 % reduction in HN30/si*JUNB*#1 cells (18.4 ± 5.2) invasiveness when compared to control (98.1 ± 27.2, *P* = 0.011) as shown in Fig. 2b. Thus, the siRNA-mediated knockdown of JunB in KCC-T871 and HN30 cells inhibited the invasive potential of these cell lines.

The scratch assay revealed significant reductions in cell motility for KCC-T871/si*JUNB*#1 cells (62.3 % ± 5.8 % vs. 83.6 % ± 4.4 %, *P* = 0.005) and KCC-T871/si*JUNB*#2 cells (8.25 % ± 2.9 % vs. 34.6 % ± 2.5 %, *P* < 0.0001) compared to control. Significant reductions in cell motility for HN30/si*JUNB*#1 cells (16.2 % ± 5.9 % vs. 92.2 % ± 2.4 %, *P* < 0.0001) and HN30/si*JUNB*#2 cells (60.9 % ± 2.9 % vs. 85.6 % ± 2.3 %, *P* < 0.0001) compared to control were also observed. Thus, the siRNA-mediated knockdown of JunB in KCC-T871 and HN30 cells also inhibited the cell migration ability as shown in Fig. 2c.

**Fig. 3:**
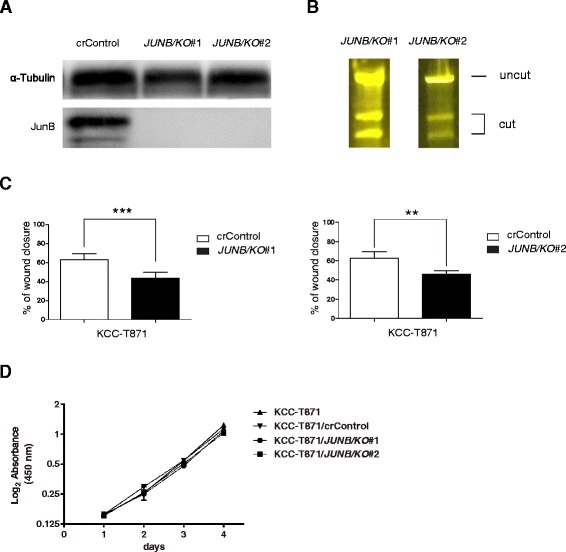
sgRNA knockout of JunB in metastatic HNSCC cells suppresses tumor migration but not cell proliferation. **a** Western blotting analysis of KCC-T871 cells transfected with control sgRNA or *JUNB* sgRNA (#1 and #2). **b** The confirmation of genome-editing in KCC-T871 cells transfected with sgRNA-mediated JunB knockout. The efficiency of sgRNAs targeting JunB was measured using a GeneArt Genomic Cleavage Detection kit. **c** sgRNA knockout JunB in KCC-T871 cells inhibited cell migration. Columns, mean % of wound closure; bars, SEM. **P < 0.01 ***P < 0.001. **d** The cell proliferation ability of KCC-T871, KCC-T871/crControl, KCC-T871/*JUNB/KO*#1 and KCC-T871/*JUNB/KO*#2 were all similar in the cell viability assay

To confirm that JunB knockdown decreased cell motility and invasiveness in HNSCC cells, the JunB knockout cells were established with two independent sgRNAs (*JUNB/KO*#1 and #2) using the CRISPR/cas9 system and the knockout was then confirmed as shown in Fig. 3a and b. As shown in Fig. 3c, the scratch assay showed significant reductions in cell motility for KCC-T871/*JUNB/KO*#1 cells (29.3 % ± 1.0 %) and KCC-T871/*JUNB/KO*#2 cells (34.2 % ± 2.1 %) compared to the control (58.1 % ± 2.5 %, *P* < 0.0001 and *P* < 0.0001, respectively), which was consistent with our previous results. These results suggested that JunB could promote HNSCC cell migration and invasion. On the other hand, the cell proliferation ability of KCC-T871, KCC-T871/crControl, KCC-T871/*JUNB/KO*#1 and KCC-T871/*JUNB/KO*#2 were similar in cell viability assays (Fig. 3d).

As JunB has been reported to contribute transforming growth factor-β-induced EMT [22], we next examined if the role of JunB in HNSCC cell migration and invasiveness is related to EMT. KCC-T871 cells showed mesenchymal characteristics at baseline as well as E-cadherin expression. Their cell morphology did develop any epithelial characteristics upon siRNA-mediated JunB knockdown in KCC-T871 cells with or without TGF-β stimulation. In addition, no phenomena associated with the down-regulation of mesenchymal markers and up-regulation of epithelial markers were observed with or without TGF-β stimulation as shown in Additional file 5: Figure S3. Furthermore, we did not observe any change in cell morphology with the down-regulation of mesenchymal markers and up-regulation of epithelial markers in response to sgRNA-mediated JunB knockout in KCC-T871 cells (data not shown).

### Knockout of JunB in metastatic HNSCC cells reduced the incidence of lung metastasis *in vivo*

**Fig. 4:**
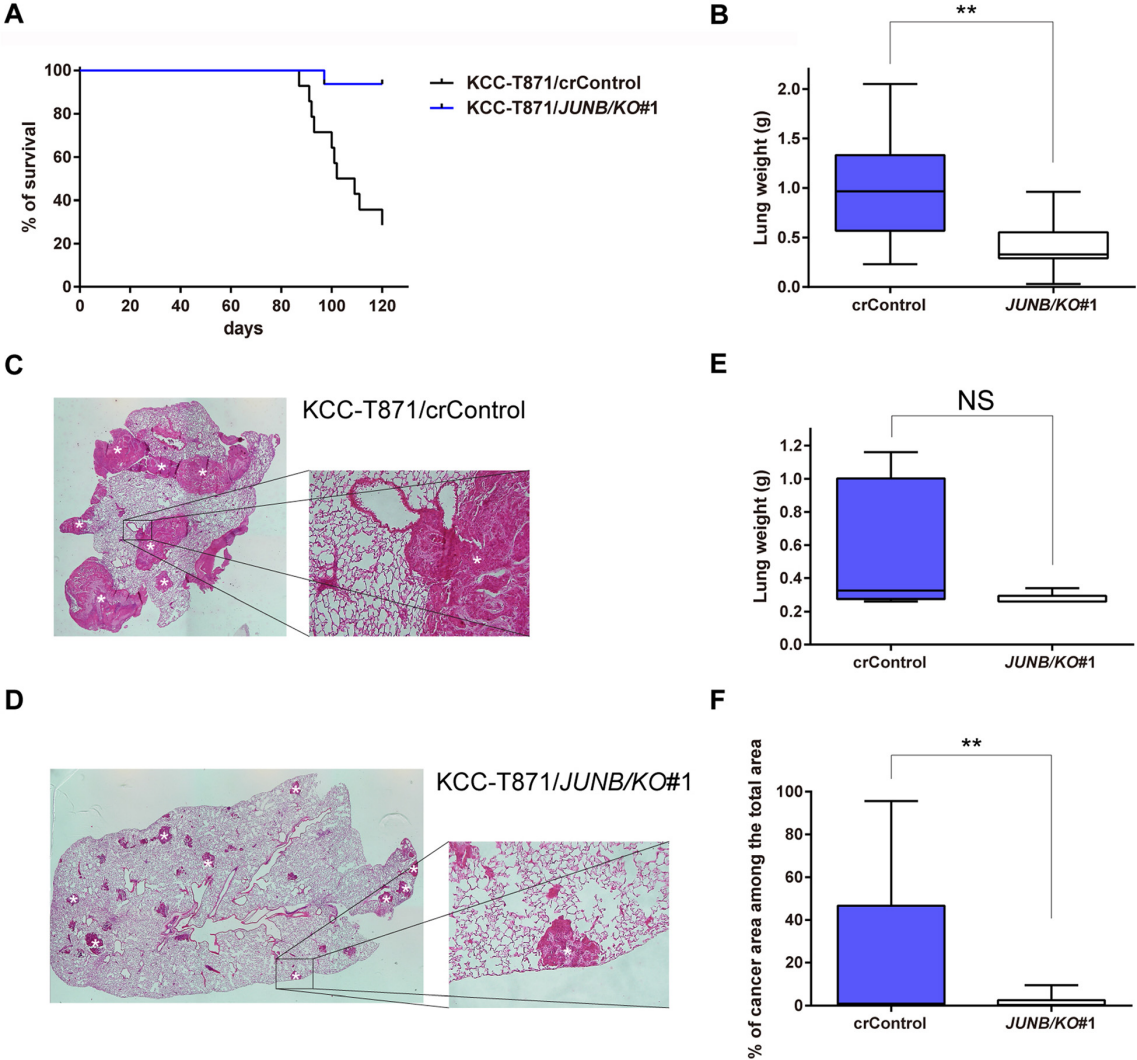
Lung metastasis was reduced in *JUNB*-knockout KCC-T871 cells in the experimental lung metastatic mouse model. **a** Survival time of the control group with KCC-T871/crControl (*N* = 14) and JunB knockout (KO) group with KCC-T871/*JUNB/KO*#1 (*N* = 16). The survival rate for the control group was significantly lower than that for the JunB KO group. ***P < 0.001. **b** Median lung weight of the control and JunB KO groups. Median lung weight of mice in the control group was significantly heavier than for that for the JunB KO group. *P* = 0.001, Boxes, 25th, 50th and 75th percentiles; bars, 10th and 90th percentiles. **c** Hematoxylin and eosin (H&E) slides of the lung from a mouse injected with KCC-T871/crControl cells in the locations marked with asterisks (*). **d** H&E slides of the lung in a mouse injected with KCC-T871/*JUNB/KO#1* cells in the locations marked with asterisks (*). **e** Median lung weight of the control group (*N* = 6) and JunB KO group (*N* = 8) in the repeated animal study. JunB knockout reduced lung weight in the animal model; however, the difference was not significant. *P* = 0.0727, Boxes, 25th, 50th and 75th percentiles; bars, 10th and 90th percentiles. **f** The area occupied by metastatic HNSCC cells in the lung in an experimental lung metastatic mouse model. The mean percentage of the lung area displaying metastatic HNSCC cells in the JunB KO group (*N* = 24) was significantly reduced compared to that in the control group (*N* = 18). **P < 0.01, Boxes, 25th, 50th and 75th percentiles; bars, 10th and 90th percentiles

To clarify the role of JunB in cell migration and invasiveness in HNSCC *in vivo*, the effect of knocking out of JunB was examined using an experimental lung metastatic mouse model. Fourteen mice for the control group with KCC-T871/crControl and 16 mice for the JunB knockout (KO) group with KCC-T871/*JUNB/KO*#1 were prepared for the survival study. The median survival period for the JunB KO group (120.0 days) was significantly greater than that for the control group (105.5 days*, P* = 0.0002, Fig. 4a). The presence of microscopic lung metastasis lesions was also impacted as 100 % of control animals had lung metastases, while 75.0 % of animals in the JunB KO group were found to have lung metastases as shown in Additional file 6: Figure S4a (*P* = 0.1029).

To confirm that JunB knockout reduced the incidence of lung metastasis in the animal model, the *in vivo* study was repeated to measure the weight of each lung and the area occupied by metastatic HNSCC cells in the lung at 78 days after cell inoculation (6 mice were used for the control group and 8 mice for the JunB KO group). Microscopic lung metastasis was detected in 83.3 % of the control mice and 62.5 % of the JunB KO mice (*P* = 0.209, Additional file 6: Figure S4b). Median lung weight was 0.325 g for the control group (0.26–1.16 g) and 0.26 g for JunB KO group (0.26–0.34 g) as shown in Fig. 4e. Thus, JunB knockout markedly reduced lung weight in the animal model; however, the difference was not significant (*P* = 0.0727). On the other hand, we observed that JunB knockout significantly reduced area occupied by metastatic HNSCC cells in the lung in the experimental lung metastatic mouse model (Additional file 7: Figure S5). As shown in Fig. 4f, the mean percentage of metastatic HNSCC cells in the lung was 21.4 % for the control group (0–95.6 %) and 1.6 % for the JunB KO group (0–9.4 %, *P* = 0.0037). Overall, we observed that knockout of JunB in metastatic HNSCC cells markedly reduced the incidence of lung metastasis in an experimental lung metastatic mouse model.

## Discussion

In this study, we identified AP-1 family as key molecules regulating the pathways related to distant metastasis in HNSCC using the upstream and key node analysis together with whole gene microarray analysis based on the *in vivo* metastatic potential of HNSCC cell lines. Among the AP-1 family, we determined that the knockdown and knockout of JunB in HNSCC cells significantly inhibited their invasion and migration *in vitro* as well as the incidence of lung metastases *in vivo*. However, no phenomena related to mesenchymal-to-epithelial transition (MET), including the depletion of morphological mesenchymal characteristics and mesenchymal markers, were observed in response to the knockdown or knockout of JunB in metastatic HNSCC cells. These results suggested that JunB could play an important role in promoting cell invasion, migration and distant metastasis in HNSCC *via* pathways other than EMT.

While there were several candidate genes related to the regulation of the pathways for distant metastasis in HNSCC identified by our key node analysis, as shown in Table 3, the AP-1 family genes were one of handful genes with high scores in the analysis for both the current dataset for distant metastatic vs. non-metastatic and our previous dataset for regional metastatic vs. non-metastatic. We previously characterized the regional metastatic potential *in vivo* using HNSCC cell lines in an orthotopic nude mouse model of HNSCC [21], and performed the upstream and key node analysis using almost the same method as that in the present study, and found that JunB and c-Fos also had high scores (data not shown). Thus, our results revealed that the AP-1 family, including JunB, might be important for regulating the pathways related not only to distant but also to regional metastasis in HNSCC.

It is well known that EMT is crucial for cancer cells not only in regard to tumor invasion and metastasis ability but also in the acquisition of resistance to apoptosis and stemness properties [23]. During the EMT process, cancer cells acquire mesenchymal characteristics instead of losing epithelial features, and increased cell migration and invasiveness is induced by stimuli or cytokines including TGF-β. Recently, several studies showing the contribution of the AP-1 family to the EMT process have been reported for several malignancies [24–26]. In fact, genome-wide profiling of AP-1–regulated transcription has revealed that c-Jun and FosL1 promote cell invasion through the repression of E-cadherin expression by the transcriptional upregulation of ZEB2 in triple-negative breast cancer cells [24]. FosL1/AP-1 signaling has also been reported to modulate ZEB1/2 and TGF-β expression to induce EMT in triple-negative breast cancer cells [24]. Moreover, cooperation between Twist1 and AP-1 has been reported to regulate integrin α5 expression to induce cell invasion by EMT [25]. Thus, AP-1 is closely associated with the EMT process in promoting the invasion and metastasis of cancer cells. However, contrary to these previous reports, we did not observe any phenomena related to MET with or without TGF-β in response to the depletion of JunB in HNSCC cells. Among the AP-1 family members, only a few studies have sought to determine the contribution of JunB to EMT, suggesting that the role of JunB in regulating EMT might be less important than that of either c-Jun and/or FosL1. There is also the possibility that the contribution of AP-1 signaling to EMT in the metastatic process in HNSCC could be relatively low compared to those for other malignancies, as the greater number of gene mutations existing in HNSCC cells, due to a history of tobacco and/or alcohol use, could play an important role in HNSCC metastasis [27].

Other mechanisms underlying the AP-1-mediated regulation of tumor invasion in cancer cells have been also reported. Kanno et al. reported that JunB regulates several genes, such as matrix metalloproteinase-2 (MMP-2), MMP-9 and chemokine (C-C motif) ligand-2 (CCL2), to promote tumor invasion and angiogenesis in VHL-defective renal cell carcinomas [28]. The AP-1/NFAT4 complex has also been reported to regulate the inhibition of E-cadherin expression by microRNA-23a during Fas-induced EMT in gastrointestinal cancer [29]. Ding et al. have identified KDM4A (lysine-specific demethylase 4A) as a key epigenetic factor activating JUN and FOSL1 to promote tumor invasion and cervical lymph node metastasis in HNSCC [12]. Thus, there are a number of mechanisms related to the regulation of tumor invasiveness by AP-1 in cancer cells. Further study is required to examine the details of the cellular and molecular mechanisms underlying the JunB-mediated promotion of tumor invasion in HNSCC.

Another limitation in the present study is that we used an experimental lung metastatic mouse model with tail vein injection of HNSCC to characterize the *in vivo* metastatic potential of HNSCC, following the identification of the AP-1 family as key molecules related to distant metastasis in HNSCC by upstream and key node analysis. The mouse model was also used to confirm the role of JunB knockout in tumor metastasis in this study. Although an experimental lung metastatic mouse model with tail vein injection of cancer cells has been widely used for studying distant metastatic potential, this model may not adequately mimic human metastatic cancer because of discrepancies in the host microenvironment in terms of the biological metastatic procedure and the absence of the cross-talk between primary and metastatic lesions [30, 31]. Basically, an orthotopic mouse model is a more adequate model with which to mimic tumor metastasis *in vivo*, as the orthotopic mouse model has advantages in terms of its ability to mimic local tumor growth and recapitulate the pathways of metastasis through a more comparable host microenvironment [32]. However, it is generally difficult to observe the development of distant metastasis in an orthotopic xenograft model of HNSCC, since the tongue tumors in the model do not allow enough time for distant metastatic lesions to develop biologically from the primary tumor generated by orthotopic implantation. Moreover, Rashid et al. have reported that an experimental lung metastatic mouse model with tail vein injection could produce lung metastatic lesion with similar genomic profiles as lung metastases after orthotopic implantation [33]. An experimental lung metastatic mouse model with tail vein injection of HNSCC was therefore used to elucidate the key molecules regulating the pathways related to metastasis in HNSCC in this study.

## Conclusions

We have identified the AP-1 family as the key molecules regulating the pathways related to distant metastasis in HNSCC by use of upstream and key nodes analysis conducted in combination with the characterization of the *in vivo* distant metastatic potential of 26 different of HNSCC cell lines in an experimental lung metastatic mouse model. The knockdown and knockout of JunB reduced tumor migration and invasion *in vitro* as well as lung metastasis *in vivo*, suggesting that the JunB pathway might be a useful a therapeutic target for inhibiting distant metastasis in patients with HNSCC. However, we did not observe any phenomena related to MET in response to JunB knockdown in HNSCC cells. Further studies are required to examine the details of the cellular and molecular mechanisms of the promotion tumor invasion by JunB in metastatic HNSCC in order to identify specific JunB inhibitors and demonstrate their efficacy in inhibiting tumor invasion and metastasis in HNSCC.

### Availability of data and materials

The GEO accession number for the agilent gene expression profiling data reported in the present study is GSE67275.

## Additional files

Additional file 1: Table S1. Primary site, source, and clinical features of tumors used to derive twenty-six HNSCC cell lines used in this study. (DOCX 20 kb) Additional file 2: Table S2. Survival, macroscopic and microscopic lung metastases, and lung weight of mice in the mouse model. (DOCX 71 kb) Additional file 3: Figure S1. Survival curves for mice injected with each of the 26 HNSCC cell lines. Animals were asphyxiated when they had lost more than 15 % of their initial body weight or had become moribund, and the remaining mice were asphyxiated 90 days after cell injection. Survival was analyzed by the Kaplan–Meier method. (PDF 488 kb) Additional file 4: Figure S2. Correlation of mean survival time with incidence of lung metastasis in an experimental lung metastatic mouse model of HNSCC. Inverse correlation between mean survival time and the incidence of lung metastasis in the mouse model was observed (*r* = –0.5192, *P* = 0.0015). (PDF 223 kb) Additional file 5: Figure S3. Cell morphology and expression of mesenchymal or epithelial marker on siRNA control or siRNA mediated JunB knockdown in KCC-T871 and HN30 cells. **a** Cell morphology of KCC-T871/siRNA control, KCC-T871/si*JUNB*#1 and KCC-T871/si*JUNB*#2. **b** Cell morphology of HN30/siRNA control, HN30/si*JUNB*#1 and HN30/si*JUNB*#2. **c** Expression of mesenchymal or epithelial marker on siRNA control or siRNA mediated JunB knockdown in KCC-T871 and HN30 cells. (PDF 1800 kb) Additional file 6: Figure S4. The incidence of microscopic lung metastasis in an experimental lung metastatic mouse model of HNSCC. **a** The incidence of microscopic lung metastasis of the control group (*N* = 14) and JunB KO group (*N* = 16). The incidence of lung metastasis in the JunB KO group (75.0 %) was reduced compared to that in the control group (100 %), however, the difference was not significant. *P* = 0.1029. **b** The incidence of microscopic lung metastasis of the control group (*N* = 6) and JunB KO group (*N* = 8) in the repeated animal study. The incidence of lung metastasis in the JunB KO group (62.5 %) was reduced compared to that in the control group (83.3 %), however, the difference was not significant. *P* = 0.5804. **c** The incidence of total lung metastasis the control group (*N* = 20) and JunB KO group (*N* = 24) in our entire animal study. The incidence of lung metastasis in the JunB KO group (70.8 %) was remarkedly reduced compared to that in the control group (95.0 %), however, the difference was not significant. *P* = 0.0544. (PDF 246 kb) Additional file 7: Figure S5. Hematoxyilin and eosin (H&E) slides of microscopic lung metastasis in an experimental lung metastatic mouse model of HNSCC. H&E slides of lung in the mouse injected with KCC-T871/crControl or KCC-T871/*JUNB/KO*#1 euthanized after 78 days following cell inoculation. A. Lung sections in the mouse injected with KCC-T871/crControl cells. B. Lung sections in the mouse injected with KCC-T871/*JUNB/KO*#1 cells. (DOCX 905 kb)
