# Supplementary material for: JunB promotes cell invasion, migration and distant metastasis of head and neck squamous cell carcinoma
Source: J Exp Clin Cancer Res. 2016 Jan 12;35:6. doi: 10.1186/s13046-016-0284-4 (PMC4709939; doi:10.1186/s13046-016-0284-4)
Supplement: Additional file 1: Table S1. — Primary site, source, and clinical features of tumors used to derive twenty-six HNSCC cell lines used in this study. (DOCX 20 kb) [file 13046_2016_284_MOESM1_ESM.docx]

**Additional file 1: Table S1** Primary site, source, and clinical features of tumors used to derive twenty-six HNSCC cell lines

| Cell line | Primary site | Age | Sex | TNM stage | Culture media † | Primary source ¶ |
| --- | --- | --- | --- | --- | --- | --- |
| **HN4** | REC (L) | 57 | M | T2N0M0 | DMEM, 10% FBS | Dr. D. M. Easty, Ludwig Institute for Cancer Research, London |
| **HN5** | REC (OC) | 73 | M | T2N0M0 | DMEM/F12, 10% FBS | Dr. D. M. Easty, Ludwig Institute for Cancer Research, London |
| **HN30** | P | - | - | - | DMEM, 10% FBS | Dr. John Ensley, Wayne State Univ. |
| **UM-SCC-1** | REC (OC) | 73 | M | T2N0M0 | DMEM, 10% FBS | Dr. Thomas E. Carey, Univ. of Michigan |
| **UM-SCC-17A** | L | 47 | F | T1N0M0 | DMEM, 10% FBS | Dr. Thomas E. Carey, Univ. of Michigan |
| **UM-SCC-17B** | EXT (UMSCC17A) | 47 | F | T1N0M0 | DMEM, 10% FBS | Dr. Thomas E. Carey, Univ. of Michigan |
| **UM-SCC-19** | OP | 67 | M | T2N1M0 | DMEM, 10% FBS | Dr. Thomas E. Carey, Univ. of Michigan |
| **MDA686LN** | LN (MDA686TU) | 48 | M | T3N3b | DMEM, 10% FBS | Dr. Peter G. Sacks, New York Univ. |
| **MDA1386TU** | HP | 72 | M | T4N3bM0 | DMEM, 10% FBS | Dr. Peter G. Sacks, New York Univ. |
| **MDA1986LN** | LN (OC) | - | - | T2N2b | DMEM, 10% FBS | Dr. Peter G. Sacks, New York Univ. |
| **FaDu** | HP | 56 | M | - | DMEM, 10% FBS | ATCC |
| **OSC-19** | LN (OC) | 61 | M | - | DMEM, 10% FBS | HSRRB |
| **TR146** | REC (OC) | 67 | F | - | DMEM, 10% FBS | Dr. Thomas Rupniak, Imperial Cancer Research Fund, London |
| **SqCC/Y1** | OC | - | - | - | DMEM/F12, 10% FBS* | Dr. Alan C. Sartorelli, Yale Univ. |
| **PE/CA-PJ34** | OC | 60 | M | - | DMEM, 10% FBS | ECACC |
| **Detroit 562** | PE (P) | - | F | - | DMEM, 10% FBS | ATCC |
| **HSC3** | tongue | 64 | M |  | DMEM, 10% FBS | HSRRB |
| **YCU-T892** | tongue | 59 | M |  | RPMI 1640, 10% FBS | Yokohama City Univ. School of Med. |
| **YCU-MS861** | Maxillary sinus | 51 | M |  | RPMI 1640, 10% FBS | Yokohama City Univ. School of Med. |
| **YCU-M911** | OP |  | M |  | RPMI 1640, 10% FBS | Yokohama City Univ. School of Med. |
| **YCU-OR891** | Oral floor | 74 | M |  | RPMI 1640, 10% FBS | Yokohama City Univ. School of Med. |
| **YCU-M862** | OP | 52 | M |  | RPMI 1640, 10% FBS | Yokohama City Univ. School of Med. |
| **KCC-T873** | Tongue |  |  |  | RPMI 1640, 10% FBS | Kanagawa Cancer Center |
| **KCC-T871** | Tongue | 56 | M |  | RPMI 1640, 10% FBS | Kanagawa Cancer Center |
| **KCC-L871** | L |  |  |  | RPMI 1640, 10% FBS | Kanagawa Cancer Center |
| **KCC-M871** | OP |  |  |  | RPMI 1640, 10% FBS | Kanagawa Cancer Center |

Abbreviations: ATCC, American Type Culture Collection; ECACC, European Collection of Cell Cultures, Wiltshire, United Kingdom; DMEM, Dulbecco’s modified Eagle’s medium; EXT, extension to adjacent tissue; FBS, fetal bovine serum; HP, hypopharynx; HSRRB, Health Science Research Resource Bank (Japan Health Sciences Foundation), Osaka, Japan; L, larynx; LN, lymph node; MDACC, The University of Texas MD Anderson Cancer Center; OC, oral cavity; OP, oropharynx; P, pharynx; PE, pleural effusion; REC, recurrence; Univ., University; UM-SCC, University of Michigan Squamous Cell Carcinoma.

† Media conditions: FBS (Sigma, St. Louis, MO). DMEM (Gibco, Invitrogen Corporation, Carlsbad, CA). DMEM/F12, Dulbecco's modified Eagle’s medium: Nutrient Mixture F-12 (Mediatech, Inc., Herndon, VA). DMEM, 10% FBS supplemented with penicillin-streptomycin, vitamin solution (Thermo Scientific, Rockford, IL), L-glutamine, nonessential amino acids (Lonza Rockland, Inc., Rockland, ME), and sodium pyruvate (Gibco). DMEM/F12, 10% FBS supplemented with penicillin-streptomycin and L-glutamine. RPMI 1640 (Nissui, Pharmaceutical Co., Tokyo), 10% FBS supplemented with penicillin-streptomycin, sodium hydrogen carbonate, 4-(2-hydroxyethyl)-1-piperazineethanesulfonic acid (HEPES).

* DMEM/F12 low glucose medium (Mediatech Inc.) 10% FBS supplemented supplemented with penicillin-streptomycin, L-glutamine

¶, Cells were established by or purchased from these sources.
