# Supplementary material for: JunB promotes cell invasion, migration and distant metastasis of head and neck squamous cell carcinoma
Source: J Exp Clin Cancer Res. 2016 Jan 12;35:6. doi: 10.1186/s13046-016-0284-4 (PMC4709939; doi:10.1186/s13046-016-0284-4)
Supplement: Additional file 2: Table S2. — Survival, macroscopic and microscopic lung metastases, and lung weight of mice in the mouse model. (DOCX 71 kb) [file 13046_2016_284_MOESM2_ESM.docx]

Additional file: Table S2. Survival, macroscopic and microscopic lung metastases, and lung weight of mice in the mouse model.

| **Cell line** | **Mouse #** | **Survival  date (d)** | **Median  survival date (d)** | **Macroscopic lung metastases** | **Microscopic lung metastases** | **% of mice with lung metastases** | **Lung weight of mice at death (g)** |
| --- | --- | --- | --- | --- | --- | --- | --- |
| **HN30** | 1 | 77 | 70 | + | + | 100.0% | 1.0 |
|  | 2 | 77 |  | + | + |  | 1.7 |
|  | 3 | 77 |  | + | + |  | 1.4 |
|  | 4 | 44 |  | + | + |  | 1.1 |
|  | 5 | 48 |  | + | + |  | 1.0 |
|  | 6 | 77 |  | + | + |  | 0.8 |
|  | 7 | 77 |  | + | + |  | 1.3 |
|  | 8 | 59 |  | + | + |  | 1.6 |
|  | 9 | 63 |  | + | + |  | 0.7 |
|  | 10 | 48 |  | + | + |  | 0.8 |
| **YCU-T892** | 1 | 43 | 43.5 | + | + | 100.0% | 1.8 |
|  | 2 | 31 |  | + | + |  | 1.9 |
|  | 3 | 66 |  | + | + |  | 1.6 |
|  | 4 | 31 |  | + | + |  | 1.7 |
|  | 5 | 44 |  | + | + |  | 1.8 |
|  | 6 | 49 |  | + | + |  | 1.8 |
|  | 7 | 50 |  | + | + |  | 2.0 |
|  | 8 | 38 |  | + | + |  | 1.7 |
|  | 9 | 45 |  | + | + |  | 1.7 |
|  | 10 | 38 |  | + | + |  | 1.9 |
| **KCC-T871** | 1 | 49 | 51.5 | + | + | 100.0% | 1.0 |
|  | 2 | 42 |  | + | + |  | 0.8 |
|  | 3 | 44 |  | + | + |  | 0.8 |
|  | 4 | 90 |  | - | + |  | 0.2 |
|  | 5 | 63 |  | + | + |  | 0.7 |
|  | 6 | 88 |  | + | + |  | 0.3 |
|  | 7 | 48 |  | + | + |  | 1.2 |
|  | 8 | 57 |  | + | + |  | 0.5 |
|  | 9 | 49 |  | + | + |  | 0.9 |
|  | 10 | 54 |  | + | + |  | 1.0 |
| **HSC-3** | 1 | 90 | 38.5 | - | - | 90.0% | 0.2 |
|  | 2 | 32 |  | + | + |  | 0.5 |
|  | 3 | 37 |  | + | + |  | 0.6 |
|  | 4 | 38 |  | + | + |  | 0.6 |
|  | 5 | 28 |  | + | + |  | 0.4 |
|  | 6 | 80 |  | + | + |  | 0.6 |
|  | 7 | 38 |  | + | + |  | 0.4 |
|  | 8 | 39 |  | + | + |  | 0.9 |
|  | 9 | 47 |  | + | + |  | 0.5 |
|  | 10 | 50 |  | + | + |  | 0.6 |
| **UM-SCC-17A** | 1 | 90 | 90 | - | + | 80.0% | 0.2 |
|  | 2 | 90 |  | - | - |  | 0.2 |
|  | 3 | 90 |  | - | - |  | 0.1 |
|  | 4 | 90 |  | - | + |  | 0.2 |
|  | 5 | 90 |  | - | + |  | 0.2 |
|  | 6 | 90 |  | - | + |  | 0.1 |
|  | 7 | 90 |  | - | + |  | 0.2 |
|  | 8 | 90 |  | - | + |  | 0.2 |
|  | 9 | 90 |  | + | + |  | 0.2 |
|  | 10 | 90 |  | + | + |  | 0.1 |
| **FaDu** | 1 | 90 | 90 | - | + | 80.0% | 0.1 |
|  | 2 | 90 |  | - | - |  | 0.2 |
|  | 3 | 90 |  | - | + |  | 0.2 |
|  | 4 | 90 |  | - | + |  | 0.1 |
|  | 5 | 90 |  | - | - |  | 0.1 |
|  | 6 | 77 |  | - | + |  | 0.1 |
|  | 7 | 90 |  | - | + |  | 0.1 |
|  | 8 | 90 |  | - | + |  | 0.2 |
|  | 9 | 90 |  | - | + |  | 0.1 |
|  | 10 | 90 |  | - | + |  | 0.3 |
| **UM-SCC-1** | 1 | 90 | 90 | + | + | 77.8% | 0.2 |
|  | 2 | 90 |  | + | + |  | 0.3 |
|  | 3 | 90 |  | + | + |  | 0.6 |
|  | 4 | 90 |  | + | + |  | 0.2 |
|  | 5 | 90 |  | + | + |  | 0.5 |
|  | 6 | 90 |  | + | + |  | 0.4 |
|  | 7 | 90 |  | - | - |  | 0.5 |
|  | 8 | 90 |  | - | - |  | 0.2 |
|  | 9 | 90 |  | + | + |  | 0.2 |
| **YCU-OR891** | 1 | 90 | 83 | - | - | 72.7% | 0.2 |
|  | 2 | 76 |  | + | + |  | 0.6 |
|  | 3 | 78 |  | + | + |  | 0.9 |
|  | 4 | 83 |  | + | + |  | 1.2 |
|  | 5 | 90 |  | + | + |  | 1.4 |
|  | 6 | 90 |  | - | - |  | 0.2 |
|  | 7 | 90 |  | + | + |  | 1.1 |
|  | 8 | 66 |  | + | + |  | 1.2 |
|  | 9 | 76 |  | + | + |  | 0.8 |
|  | 10 | 67 |  | + | + |  | 1.1 |
|  | 11 | 90 |  | - | - |  | 0.2 |
| **KCC-T873** | 1 | 90 | 90 | - | - | 70.0% | 0.2 |
|  | 2 | 90 |  | - | - |  | 0.2 |
|  | 3 | 90 |  | + | + |  | 0.2 |
|  | 4 | 88 |  | + | + |  | 0.9 |
|  | 5 | 90 |  | - | - |  | 0.2 |
|  | 6 | 90 |  | + | + |  | 0.3 |
|  | 7 | 90 |  | + | + |  | 0.3 |
|  | 8 | 90 |  | + | + |  | 0.2 |
|  | 9 | 90 |  | + | + |  | 0.7 |
|  | 10 | 90 |  | + | + |  | 0.8 |
| **TR146** | 1 | 90 | 90 | - | + | 62.5% | 0.1 |
|  | 2 | 90 |  | - | - |  | 0.1 |
|  | 3 | 90 |  | - | + |  | 0.1 |
|  | 4 | 90 |  | - | + |  | 0.1 |
|  | 5 | 90 |  | - | + |  | 0.1 |
|  | 6 | 90 |  | - | - |  | 0.2 |
|  | 7 | 90 |  | + | + |  | 0.4 |
|  | 8 | 90 |  | - | - |  | 0.2 |
| **YCU-M911** | 1 | 90 | 90 | - | - | 60.0% | 0.2 |
|  | 2 | 77 |  | + | + |  | 0.7 |
|  | 3 | 77 |  | + | + |  | 0.5 |
|  | 4 | 90 |  | - | - |  | 0.2 |
|  | 5 | 83 |  | + | + |  | 0.2 |
|  | 6 | 90 |  | + | + |  | 0.3 |
|  | 7 | 90 |  | + | + |  | 0.4 |
|  | 8 | 90 |  | + | + |  | 0.2 |
|  | 9 | 90 |  | - | - |  | 0.2 |
|  | 10 | 90 |  | - | - |  | 0.2 |
| **MDA686LN** | 1 | 90 | 90 | - | - | 60.0% | 0.1 |
|  | 2 | 90 |  | - | + |  | 0.1 |
|  | 3 | 90 |  | - | - |  | 0.1 |
|  | 4 | 90 |  | - | + |  | 0.2 |
|  | 5 | 90 |  | - | + |  | 0.1 |
|  | 6 | 90 |  | - | - |  | 0.1 |
|  | 7 | 90 |  | - | - |  | 0.1 |
|  | 8 | 90 |  | - | + |  | 0.1 |
|  | 9 | 90 |  | - | + |  | 0.1 |
|  | 10 | 90 |  | - | + |  | 0.1 |
| **UM-SCC-17B** | 1 | 90 | 90 | - | - | 50.0% | 0.1 |
|  | 2 | 90 |  | - | - |  | 0.1 |
|  | 3 | 90 |  | - | + |  | 0.1 |
|  | 4 | 90 |  | + | + |  | 0.1 |
|  | 5 | 90 |  | + | + |  | 0.1 |
|  | 6 | 90 |  | + | + |  | 0.1 |
|  | 7 | 90 |  | - | - |  | 0.1 |
|  | 8 | 77 |  | - | - |  | 0.1 |
|  | 9 | 87 |  | + | + |  | 1.3 |
|  | 10 | 90 |  | - | - |  | 0.1 |
| **HN4** | 1 | 90 | 90 | - | - | 50.0% | 0.1 |
|  | 2 | 90 |  | + | + |  | 0.1 |
|  | 3 | 90 |  | - | + |  | 0.1 |
|  | 4 | 90 |  | + | - |  | 0.1 |
|  | 5 | 90 |  | - | + |  | 0.1 |
|  | 6 | 90 |  | - | + |  | 0.1 |
|  | 7 | 77 |  | - | - |  | 0.5 |
|  | 8 | 90 |  | - | - |  | 0.1 |
|  | 9 | 90 |  | - | - |  | 0.1 |
|  | 10 | 90 |  | - | + |  | 0.1 |
| **SqCC/Y1** | 1 | 90 | 90 | - | - | 44.4% | 0.2 |
|  | 2 | 90 |  | + | + |  | 0.2 |
|  | 3 | 90 |  | + | + |  | 0.5 |
|  | 4 | 90 |  | - | - |  | 0.2 |
|  | 5 | 90 |  | + | + |  | 0.1 |
|  | 6 | 90 |  | - | - |  | 0.1 |
|  | 7 | 90 |  | - | - |  | 0.2 |
|  | 8 | 90 |  | + | + |  | 0.2 |
|  | 9 | 90 |  | - | - |  | 0.2 |
| **UM-SCC-19** | 1 | 90 | 90 | - | - | 37.5% | 0.2 |
|  | 2 | 90 |  | - | + |  | 0.1 |
|  | 3 | 90 |  | - | - |  | 0.2 |
|  | 4 | 90 |  | - | + |  | 0.2 |
|  | 5 | 90 |  | - | - |  | 0.2 |
|  | 6 | 90 |  | - | + |  | 0.2 |
|  | 7 | 90 |  | - | - |  | 0.2 |
|  | 8 | 90 |  | - | - |  | 0.1 |
| **HN5** | 1 | 90 | 90 | - | - | 30.0% | 0.1 |
|  | 2 | 90 |  | - | + |  | 0.1 |
|  | 3 | 90 |  | - | + |  | 0.1 |
|  | 4 | 90 |  | - | - |  | 0.1 |
|  | 5 | 90 |  | - | - |  | 0.2 |
|  | 6 | 90 |  | + | + |  | 0.2 |
|  | 7 | 90 |  | - | - |  | 0.1 |
|  | 8 | 90 |  | - | - |  | 0.1 |
|  | 9 | 90 |  | - | - |  | 0.2 |
|  | 10 | 90 |  | - | - |  | 0.2 |
| **OSC-19** | 1 | 90 | 90 | - | - | 20.0% | 0.1 |
|  | 2 | 90 |  | - | + |  | 0.2 |
|  | 3 | 90 |  | - | - |  | 0.2 |
|  | 4 | 90 |  | - | - |  | 0.2 |
|  | 5 | 90 |  | - | - |  | 0.2 |
|  | 6 | 90 |  | - | - |  | 0.2 |
|  | 7 | 90 |  | - | - |  | 0.2 |
|  | 8 | 90 |  | - | + |  | 0.1 |
|  | 9 | 90 |  | - | - |  | 0.2 |
|  | 10 | 90 |  | - | - |  | 0.2 |
| **MDA1386TU** | 1 | 90 | 90 | - | + |  | 0.2 |
|  | 2 | 90 |  | - | - |  | 0.1 |
|  | 3 | 90 |  | - | - |  | 0.2 |
|  | 4 | 90 |  | - | - |  | 0.1 |
|  | 5 | 90 |  | + | + |  | 0.1 |
|  | 6 | 90 |  | - | - |  | 0.2 |
|  | 7 | 90 |  | - | - |  | 0.2 |
|  | 8 | 90 |  | - | - |  | 0.2 |
|  | 9 | 90 |  | - | - |  | 0.1 |
|  | 10 | 90 |  | - | - |  | 0.2 |
| **MDA1986LN** | 1 | 90 | 90 | - | - | 20.0% | 0.2 |
|  | 2 | 90 |  | - | - |  | 0.2 |
|  | 3 | 90 |  | - | - |  | 0.1 |
|  | 4 | 90 |  | - | - |  | 0.1 |
|  | 5 | 90 |  | - | + |  | 0.2 |
|  | 6 | 90 |  | + | + |  | 0.2 |
|  | 7 | 90 |  | - | - |  | 0.1 |
|  | 8 | 90 |  | - | - |  | 0.1 |
|  | 9 | 90 |  | - | - |  | 0.2 |
|  | 10 | 90 |  | - | - |  | 0.2 |
| **KCC-L871** | 1 | 90 | 90 | - | - | 10.0% | 0.2 |
|  | 2 | 90 |  | - | - |  | 0.2 |
|  | 3 | 90 |  | - | - |  | 0.2 |
|  | 4 | 90 |  | - | - |  | 0.2 |
|  | 5 | 90 |  | - | - |  | 0.2 |
|  | 6 | 90 |  | - | - |  | 0.2 |
|  | 7 | 90 |  | - | - |  | 0.2 |
|  | 8 | 90 |  | - | - |  | 0.2 |
|  | 9 | 90 |  | + | + |  | 0.3 |
|  | 10 | 90 |  | - | - |  | 0.2 |
| **Detroit562** | 1 | 90 | 90 | - | - | 0.0% | 0.1 |
|  | 2 | 90 |  | - | - |  | 0.1 |
|  | 3 | 62 |  | - | - |  | 0.2 |
|  | 4 | 90 |  | - | - |  | 0.2 |
|  | 5 | 90 |  | - | - |  | 0.2 |
|  | 6 | 90 |  | - | - |  | 0.2 |
|  | 7 | 90 |  | - | - |  | 0.1 |
|  | 8 | 90 |  | - | - |  | 0.1 |
|  | 9 | 90 |  | - | - |  | 0.1 |
| **PE/CA-PJ34** | 1 | 90 | 90 | - | - | 0.0% | 0.1 |
|  | 2 | 90 |  | - | - |  | 0.1 |
|  | 3 | 90 |  | - | - |  | 0.1 |
|  | 4 | 90 |  | - | - |  | 0.2 |
|  | 5 | 90 |  | - | - |  | 0.1 |
|  | 6 | 90 |  | - | - |  | 0.1 |
|  | 7 | 90 |  | - | - |  | 0.2 |
|  | 8 | 90 |  | - | - |  | 0.2 |
|  | 9 | 90 |  | - | - |  | 0.1 |
| **KCC-M871** | 1 | 90 | 90 | - | - | 0.0% | 0.2 |
|  | 2 | 90 |  | - | - |  | 0.2 |
|  | 3 | 90 |  | - | - |  | 0.2 |
|  | 4 | 90 |  | - | - |  | 0.2 |
|  | 5 | 90 |  | - | - |  | 0.2 |
|  | 6 | 90 |  | - | - |  | 0.2 |
|  | 7 | 90 |  | - | - |  | 0.2 |
|  | 8 | 90 |  | - | - |  | 0.2 |
|  | 9 | 90 |  | - | - |  | 0.2 |
|  | 10 | 90 |  | - | - |  | 0.2 |
| **YCU-MS861** | 1 | 90 | 90 | - | - | 0.0% | 0.2 |
|  | 2 | 90 |  | - | - |  | 0.2 |
|  | 3 | 90 |  | - | - |  | 0.2 |
|  | 4 | 90 |  | - | - |  | 0.2 |
|  | 5 | 90 |  | - | - |  | 0.2 |
|  | 6 | 90 |  | - | - |  | 0.2 |
|  | 7 | 90 |  | - | - |  | 0.2 |
|  | 8 | 90 |  | - | - |  | 0.2 |
|  | 9 | 90 |  | - | - |  | 0.2 |
|  | 10 | 90 |  | - | - |  | 0.2 |
| **YCU-M862** | 1 | 90 | 90 | - | - | 0.0% | 0.2 |
|  | 2 | 90 |  | - | - |  | 0.2 |
|  | 3 | 90 |  | - | - |  | 0.2 |
|  | 4 | 90 |  | - | - |  | 0.2 |
|  | 5 | 90 |  | - | - |  | 0.2 |
|  | 6 | 90 |  | - | - |  | 0.2 |
|  | 7 | 90 |  | - | - |  | 0.2 |
|  | 8 | 90 |  | - | - |  | 0.2 |
|  | 9 | 90 |  | - | - |  | 0.2 |
|  | 10 | 91 |  | - | - |  | 0.2 |
|  | 11 | 90 |  | - | - |  | 0.2 |
