# Supplementary figures and images for: JunB promotes cell invasion, migration and distant metastasis of head and neck squamous cell carcinoma
Source: J Exp Clin Cancer Res. 2016 Jan 12;35:6. doi: 10.1186/s13046-016-0284-4 (PMC4709939; doi:10.1186/s13046-016-0284-4)

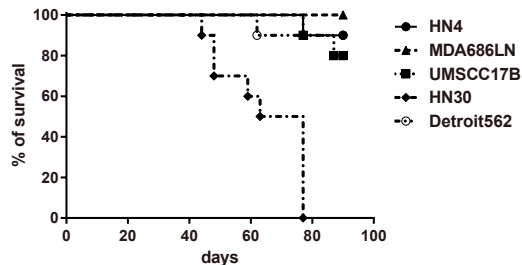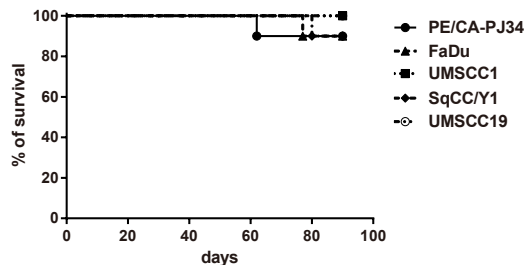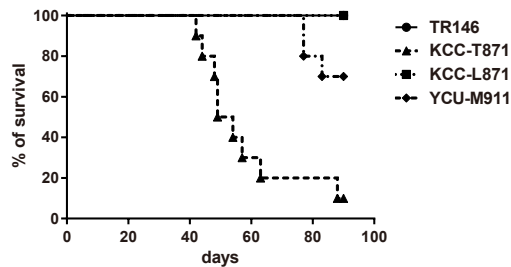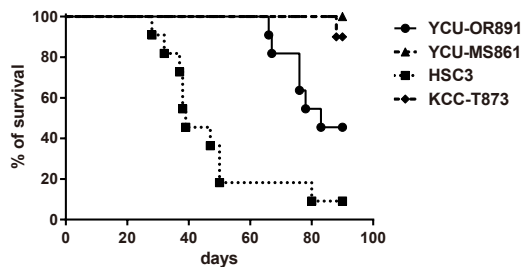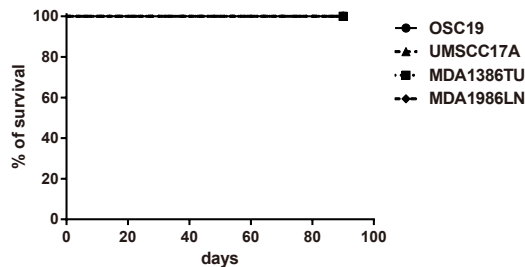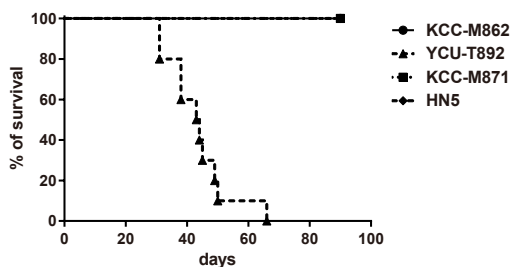

Supplement: Additional file 3: Figure S1. — Survival curves for mice injected with each of the 26 HNSCC cell lines. Animals were asphyxiated when they had lost more than 15 % of their initial body weight or had become moribund, and the remaining mice were asphyxiated 90 days after cell injection. Survival was analyzed by the Kaplan–Meier method. (PDF 488 kb) [file 13046_2016_284_MOESM3_ESM.pdf]

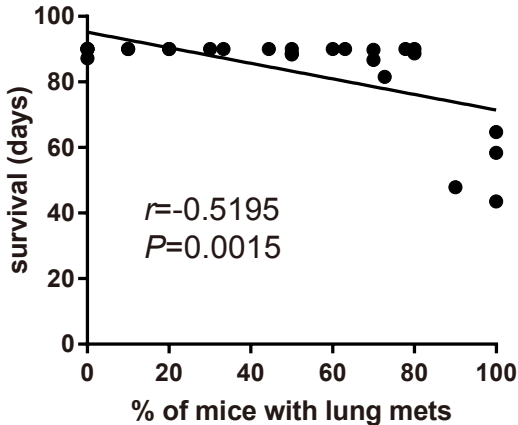

Supplement: Additional file 4: Figure S2. — Correlation of mean survival time with incidence of lung metastasis in an experimental lung metastatic mouse model of HNSCC. Inverse correlation between mean survival time and the incidence of lung metastasis in the mouse model was observed (r = –0.5192, P = 0.0015). (PDF 223 kb) [file 13046_2016_284_MOESM4_ESM.pdf]

**A**

KCC-T871

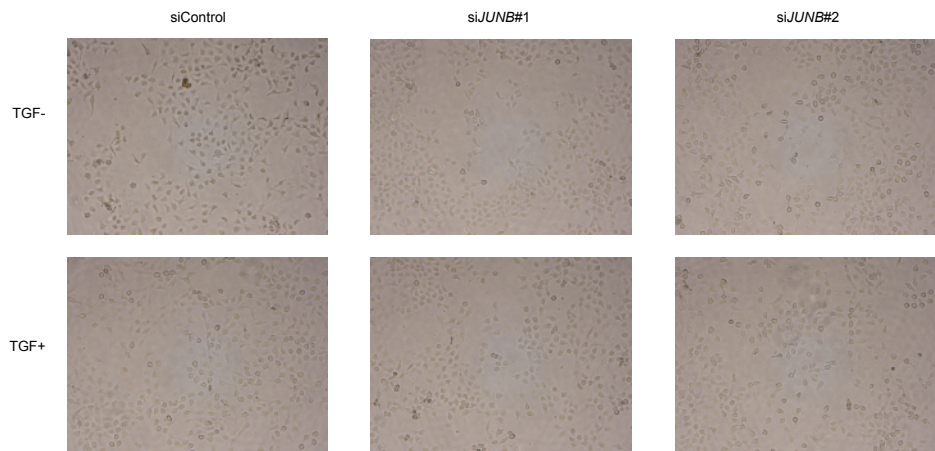**B**

HN30

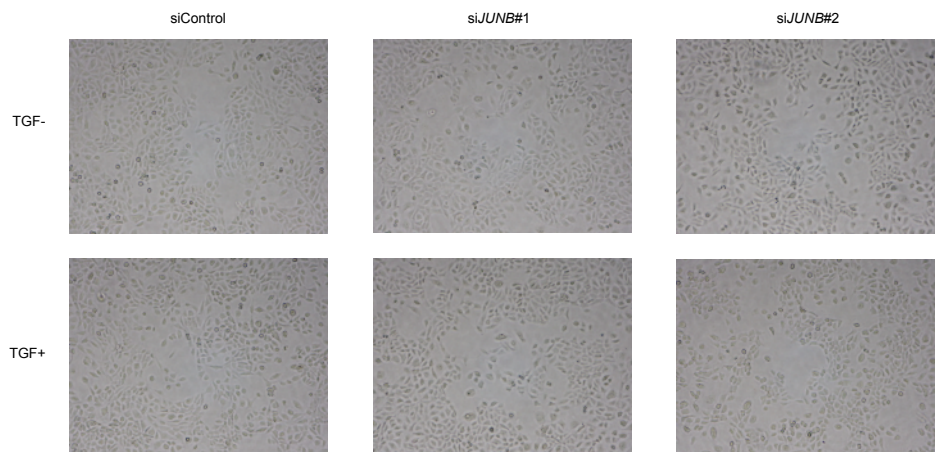**C**

KCC-T871

HN30

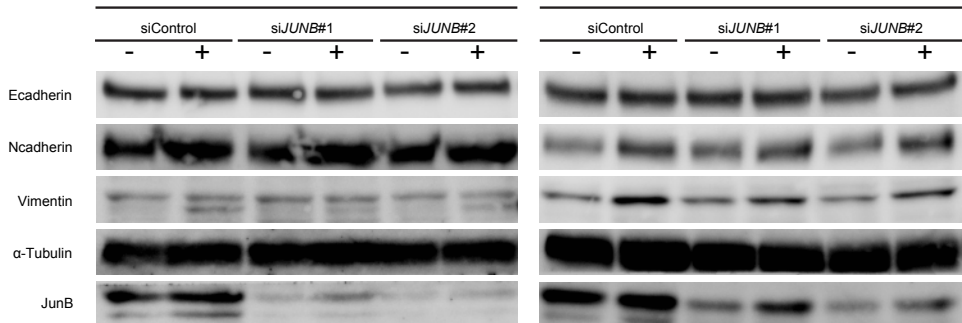

Supplement: Additional file 5: Figure S3. — Cell morphology and expression of mesenchymal or epithelial marker on siRNA control or siRNA mediated JunB knockdown in KCC-T871 and HN30 cells. a Cell morphology of KCC-T871/siRNA control, KCC-T871/siJUNB#1 and KCC-T871/siJUNB#2. b Cell morphology of HN30/siRNA control, HN30/siJUNB#1 and HN30/siJUNB#2. c Expression of mesenchymal or epithelial marker on siRNA control or siRNA mediated JunB knockdown in KCC-T871 and HN30 cells. (PDF 1800 kb) [file 13046_2016_284_MOESM5_ESM.pdf]

**A**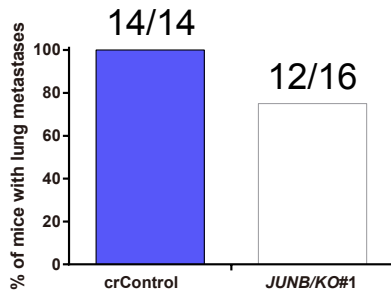**B**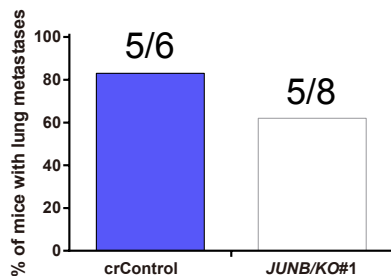**C**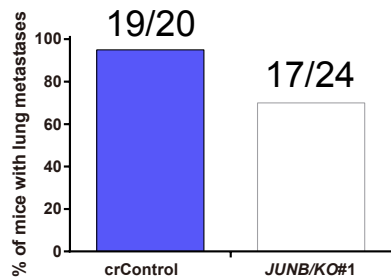

Supplement: Additional file 6: Figure S4. — The incidence of microscopic lung metastasis in an experimental lung metastatic mouse model of HNSCC. a The incidence of microscopic lung metastasis of the control group (N = 14) and JunB KO group (N = 16). The incidence of lung metastasis in the JunB KO group (75.0 %) was reduced compared to that in the control group (100 %), however, the difference was not significant. P = 0.1029. b The incidence of microscopic lung metastasis of the control group (N = 6) and JunB KO group (N = 8) in the repeated animal study. The incidence of lung metastasis in the JunB KO group (62.5 %) was reduced compared to that in the control group (83.3 %), however, the difference was not significant. P = 0.5804. c The incidence of total lung metastasis the control group (N = 20) and JunB KO group (N = 24) in our entire animal study. The incidence of lung metastasis in the JunB KO group (70.8 %) was remarkedly reduced compared to that in the control group (95.0 %), however, the difference was not significant. P = 0.0544. (PDF 246 kb) [file 13046_2016_284_MOESM6_ESM.pdf]

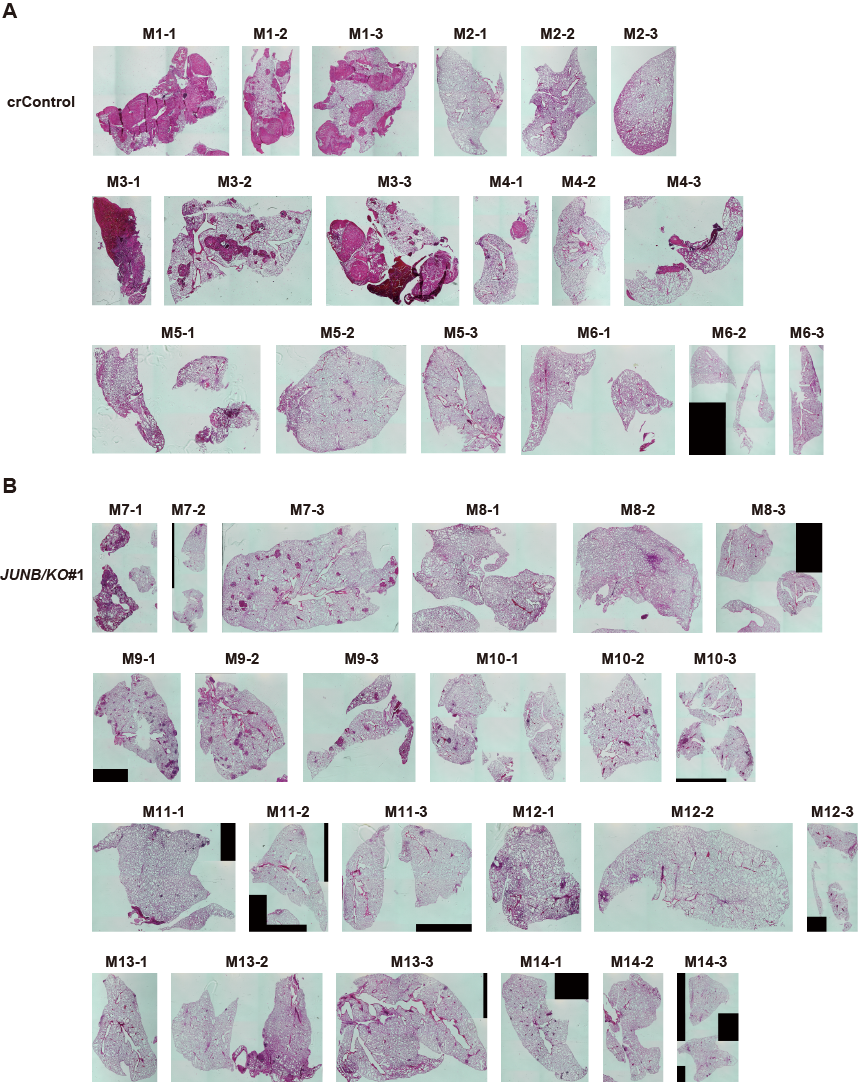

Supplement: Additional file 7: Figure S5. — Hematoxyilin and eosin (H&E) slides of microscopic lung metastasis in an experimental lung metastatic mouse model of HNSCC. H&E slides of lung in the mouse injected with KCC-T871/crControl or KCC-T871/JUNB/KO#1 euthanized after 78 days following cell inoculation. A. Lung sections in the mouse injected with KCC-T871/crControl cells. B. Lung sections in the mouse injected with KCC-T871/JUNB/KO#1 cells. (DOCX 905 kb) [file 13046_2016_284_MOESM7_ESM.docx]
